# Human group coordination in a sensorimotor task with neuron-like decision-making

**DOI:** 10.1038/s41598-020-64091-4

**Published:** 2020-05-19

**Authors:** Gerrit Schmid, Daniel A. Braun

**Affiliations:** Faculty of Engineering, Computer Science and Psychology, Institute of Neural Information Processing, Ulm University, 89081 Ulm, Germany

**Keywords:** Computational models, Computational neuroscience, Motor control

## Abstract

The formation of cooperative groups of agents with limited information-processing capabilities to solve complex problems together is a fundamental building principle that cuts through multiple scales in biology from groups of cells to groups of humans. Here, we study an experimental paradigm where a group of humans is joined together to solve a common sensorimotor task that cannot be achieved by a single agent but relies on the cooperation of the group. In particular, each human acts as a neuron-like binary decision-maker that determines in each moment of time whether to be active or not. Inspired by the population vector method for movement decoding, each neuron-like decision-maker is assigned a preferred movement direction that the decision-maker is ignorant about. From the population vector reflecting the group activity, the movement of a cursor is determined, and the task for the group is to steer the cursor into a predefined target. As the preferred movement directions are unknown and players are not allowed to communicate, the group has to learn a control strategy on the fly from the shared visual feedback. Performance is analyzed by learning speed and accuracy, action synchronization, and group coherence. We study four different computational models of the observed behavior, including a perceptron model, a reinforcement learning model, a Bayesian inference model and a Thompson sampling model that efficiently approximates Bayes optimal behavior. The Bayes and especially the Thompson model excel in predicting the human group behavior compared to the other models, suggesting that internal models are crucial for adaptive coordination. We discuss benefits and limitations of our paradigm regarding a better understanding of distributed information processing.

## Introduction

Multi-agent cooperation in uncertain environments is a pervasive phenomenon in the biological sciences ranging from the cooperation of simple molecules, the cooperation between cells both in microbes and in multicellular organisms, the cooperation between cell conglomerates or tissues making up organs, to the cooperation between multiple organisms forming groups^1–4^. As a consequence of joining individuals together into groups, complex behavior can emerge from the interaction and cooperation of a multitude of relatively simple decision-makers. Without doubt the brain is one of the most astonishing examples of such an emergence, where billions of nerve cells cooperate to produce complex sensorimotor coordination. If we regard each individual neuron as an elementary decision-maker, each of these decision-makers would be rather limited in the information it can process, but many neurons together may be able to solve complicated information-processing problems beyond the capabilities of each individual. Similarly, we may regard groups of humans (e.g. a business organization) as distributed decision-makers, where each individual human has rather limited information-processing capabilities, but as a group they may solve complex problems beyond the abilities of each individual^5–9^.

In the social sciences, decision-making in groups is often studied in formal frameworks like social choice theory, consensus reaching processes and voting-based methods, where the aim is to balance the preferences of different decision-makers. For example, it has been proposed that iterative decision-making processes using feedback and gamification can be useful tools to achieve such a balance^10^. The study of group decision-making is a particular application of game theory that investigates general interactions between multiple individuals^11^. Game theory can be subdivided into the two branches of cooperative and non-cooperative game theory. Non-cooperative game theory presupposes that each individual optimizes their own utility, which in some cases can lead to mutualisms, and in some cases to direct competition, especially in so-called zero-sum games where one player’s loss is the other player’s gain (e.g. board games like chess or checkers). The optimal solution in a non-cooperative game typically takes the form of a Nash equilibrium, where each player chooses a strategy such that neither player has anything to gain by deviating from this strategy^12–14^. In contrast, cooperative game theory assumes a joint payoff that is distributed amongst all participating players and the problem is which players form coalitions and how to allocate the reward. Accordingly, the solution to a cooperative game consists of a payoff allocation vector^15–17^.

Non-cooperative game theory has been previously applied to dyadic sensorimotor interactions where players were not explicitly made aware of playing particular games (e.g. they were not told the story of the prisoners’ dilemma), but simply exposed to haptic interfaces with coupled dynamics in which they tried to minimize forces^18–20^. It was found that human players in such sensorimotor games acted in line with predicted game-theoretic solutions, whereas players in the cognitive version of the game often do not. In particular, it was found that in the sensorimotor version of the prisoners’ dilemma players’ behavior was predicted by the Nash equilibrium, even though in cognitive versions of the game it has been frequently reported that humans cooperate in direct violation of the Nash assumption^19^. The same kind of setup has also been extended to study sensorimotor coordination games^18^, where players have to select between multiple Nash equilibria, and to investigate sensorimotor Bayesian games^20^, where one player has to infer the type of another player from the other player’s signals. The important feature of all these sensorimotor games is that the Nash equilibrium is not arrived at by deliberation, but by sensorimotor learning leading to stable dynamics.

Cooperative game theory has a focus on cooperation in larger groups, for example in coalitional skill games where each agent has a set of skills required to complete a number of tasks and the question is which agents to include in each task^21^. However, experimental investigations of cooperative game theory with larger groups in the sensorimotor domain are so far limited. For instance, social loafing has been reported in cooperative tasks like tug-of-war^22^, where a group of people pulls on a rope, but some participants pull less than they could at the expense of others. While tug-of-war certainly poses a cooperation problem that cannot be solved by any individual alone, all players essentially do the same thing, and so the game misses out one of the most important aspects of cooperation in large groups, namely specialization. It is through specialization that groups of limited individuals become efficient, for example dedicated neurons that become sensitive to particular features of the environment^23–25^, or human experts that are able to devote their attention and skills to very particular problems^26,27^.

In our study we investigate groups of humans together solving a sensorimotor task, where each individual contributes a different skill. Unlike the simple game of tug-of-war, players have to coordinate these skills and also have to learn about their skill. This is similar to the situation faced by a neuron inside a brain that does not know how its actions influence the environment. On this very abstract level, we may ask whether there are similar principles underlying the cooperation of humans and the cooperation of cells in a brain. It is in this analogy that we wish to study artificial “brains” made up of multiple human individuals with limited abilities, that together solve problems of sensorimotor coordination.

### Results

**Figure 1:**
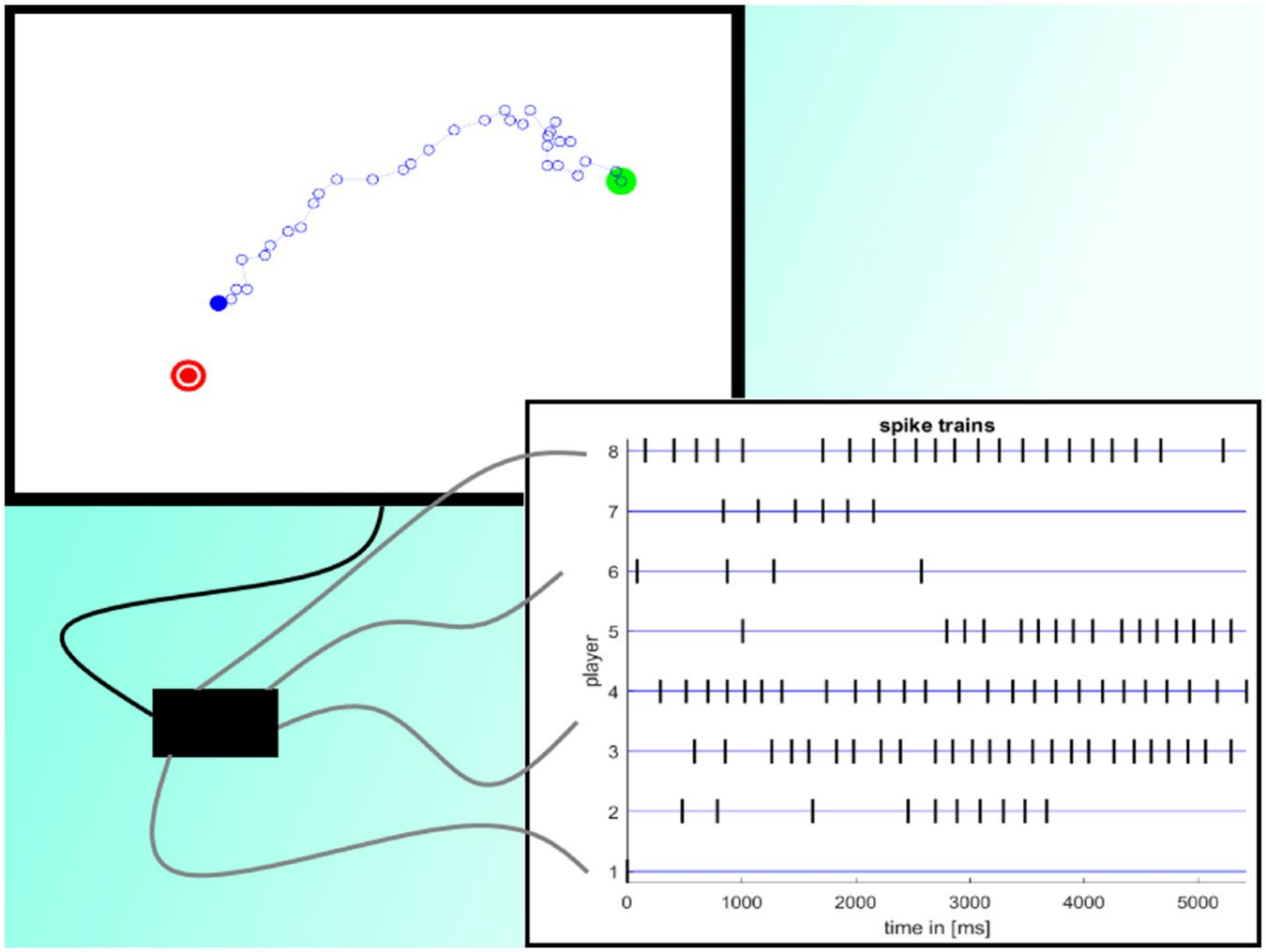
The NeuronGame. Participants sit in front of a shared screen and have to cooperate in order to steer a cursor (solid blue circle) from a start (green circle) to a goal position (red circle). Each player is assigned an unknown movement direction such that a button press of that player moves the cursor with a small step into that direction. Players are not allowed to communicate, so the difficulty is for each player to figure out their movement direction while the entire group is playing. In each instance of time each player has to decide whether to be active or inactive, this way, generating a spike train of active events. The portrayed movement pattern (blue-rimmed circles) is a typical example extracted from the experiment. Near the start, within the first two to three seconds of the level when all players try to learn the effect of their actions, the cursor’s movement seems to be erratic. As soon as players gather an adequate amount of information, some players (in the example player 1, 6, 7, 2) stop spiking while the remaining players proceed to refine the movement.

In our sensorimotor task that we dub the NeuronGame—see Fig. 1 and Section Experimental Design—eight players are equipped with a push-button each and gathered in front of a shared screen. In this game, pressing a single button produces a tiny cursor displacement on the screen in a predefined direction unknown to the player, pressing multiple buttons simultaneously simply generates the sum of these basic displacements. This way each player generates a spike train of button-press events, hence, eight spike trains in total. The difficulty faced by each player is to figure out their contribution to the movement on the fly without communication. The goal of the game is that the group of players manages to repeatedly steer the cursor from a start to a target location, where each repetition corresponds to a trial. Participants have to learn when to press their push-button as they are initially unaware of the actual effect of their actions. The learning problem is exacerbated by the uncertainty evoked through the actions of the other players and the imprecise observation of the joint movement presented on the screen. With growing certainty about the effect of their own actions, players start to cooperate, enabling the group to reach the target, which is in general not possible to achieve by the actions of a single player. To allow for multiple realizations of the learning problem, the predefined direction of displacement for each player was randomized every ten trials. In total, there were forty batches of ten trials each and we analyze the behavior of the players averaged across all batches.

Throughout the paper we compare participants’ behavior to four different computational models to allow for better interpretability of the results:

- a perceptron model (ANN) where each player corresponds to a simple threshold neuron with adaptable input weights, and where the error signal is binary indicating whether the action of a player was followed by a combined cursor displacement that either increased or decreased the cursor’s distance to the target^28^,
- a reinforcement learning model (RL) where each player corresponds to a SARSA$(\lambda )$ learner with two actions with a binary reward analogous to the ANN error signal^29^,
- a Bayes-optimal decision-making model (Bayes) where each player is assumed to hold and incrementally update a Bayesian belief $\pi$ about their individual displacement direction, and to use this belief to optimally decide whether to participate in the movement at each point in time or not,
- and a Thompson sampling model (Thompson) that efficiently approximates Bayes optimal decisions. The Thompson decision-maker holds a Bayesian belief analogous to the Bayes decision-making model, samples from this belief at every point in time and acts optimally given the sample.

Importantly, the first two models are reward-based, the last two approaches are based on the assumption of decision-makers having internal models. All models are able to solve the NeuronGame, but exhibit different learning dynamics on the way. Although the parameters of all models were fitted such that model performance is as close as possible to human performance, there is a significant difference in fitting quality when trying to predict and explain human behavior. While the ANN and RL model fail to yield overall good predictions, we find that the Bayesian model and the Thompson model provide a good fit explaining group coordination. Details of the models can be found in the methods section entitled Computational models.

### Quantifying cooperation in groups

In order to quantify the degree of cooperation in a group, we analyze all decisions made by the group and determine the proportion of productive and counterproductive decisions, which we term success and failure rate in the following. We also analyze the proportion of productive intervention of individuals. Moreover, we consider internal coherence of a group of binary actors inspired by neuronal measures of coherence^30^, in order to capture to what degree the eight players act cooperatively as a single unit. Accordingly, one would expect that players whose displacement directions are in close vicinity tend to act together, whereas players with opposing displacement directions should avoid acting at the same time. In order to quantify this coherence of spiking patterns, we look at two different measures, namely the action time correlations between spike trains of any pair of players and the event synchronization^31^ that has previously been proposed to measure the similarity between spike trains in neural recordings—see section Data analysis for details.

#### Success rate and group synergy

**Figure 2:**
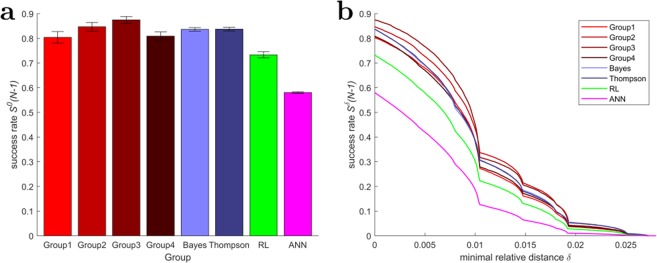
Success rate of group decisions. (**a**) Proportion of time points where actions steered the cursor closer to the target for the four human groups and the four computational models. Error bars indicate 99% confidence intervals. (**b**) Success rate as a function of the minimally required improvement $\delta$ of the cursor displacement relative to the target. The improvement threshold is measured as a dimensionless fraction of display size and ranges between $0$ and $0.028$. The minimum value naturally corresponds to the case where the cursor is not moved at all, the maximum value corresponds to the best case improvement. Large improvements (e.g. $\ge 0.02$) are rare.

In our analysis, a decision corresponds to a subset of players being active within a time interval of approximately 130 ms (see Supplementary Table S1). Such a group decision entails a cursor displacement resulting from the vector sum of individual cursor displacements. The decision is productive if the new cursor position is closer to the target than the previous cursor position. The success rate then determines the ratio of productive decisions made by an ensemble of players relative to the total number of decisions. As can be seen in Fig. 2a, the average success rate of our human groups was roughly 83%, that means in 83% of time points where at least one player was active, the group action moved the cursor closer to the target. Conversely, in 17% of time points, the group steered the cursor away from the target. The Bayesian and the Thompson model provide the closest match in success rate with 84% success rates in both models. The best-fitting RL model has a success rate of 73%, and the best-fitting perceptron model has a success rate of 58%, both distinctly below the experimental values. We can also refine our notion of success rate, by only counting those time steps as successful in which the cursor moved closer to the target by at least a minimum threshold distance. This is depicted in Fig. 2b. Again the Bayesian and Thompson model are closest to the experimental curves, the success rate of the other two models is significantly lower. One notable difference between the human group and the two best fitting models is, that for minimal distances $\delta$ that require only one action of a single player, both, the Thompson and the Bayes model perform slightly worse than the average human group, whereas for larger $\delta$ requiring at least four players cooperating—transitions requiring multiple players are marked by the little *bumps* in Fig. 2—, the Thompson and the Bayes model have superior performance over the human players.

**Figure 3:**
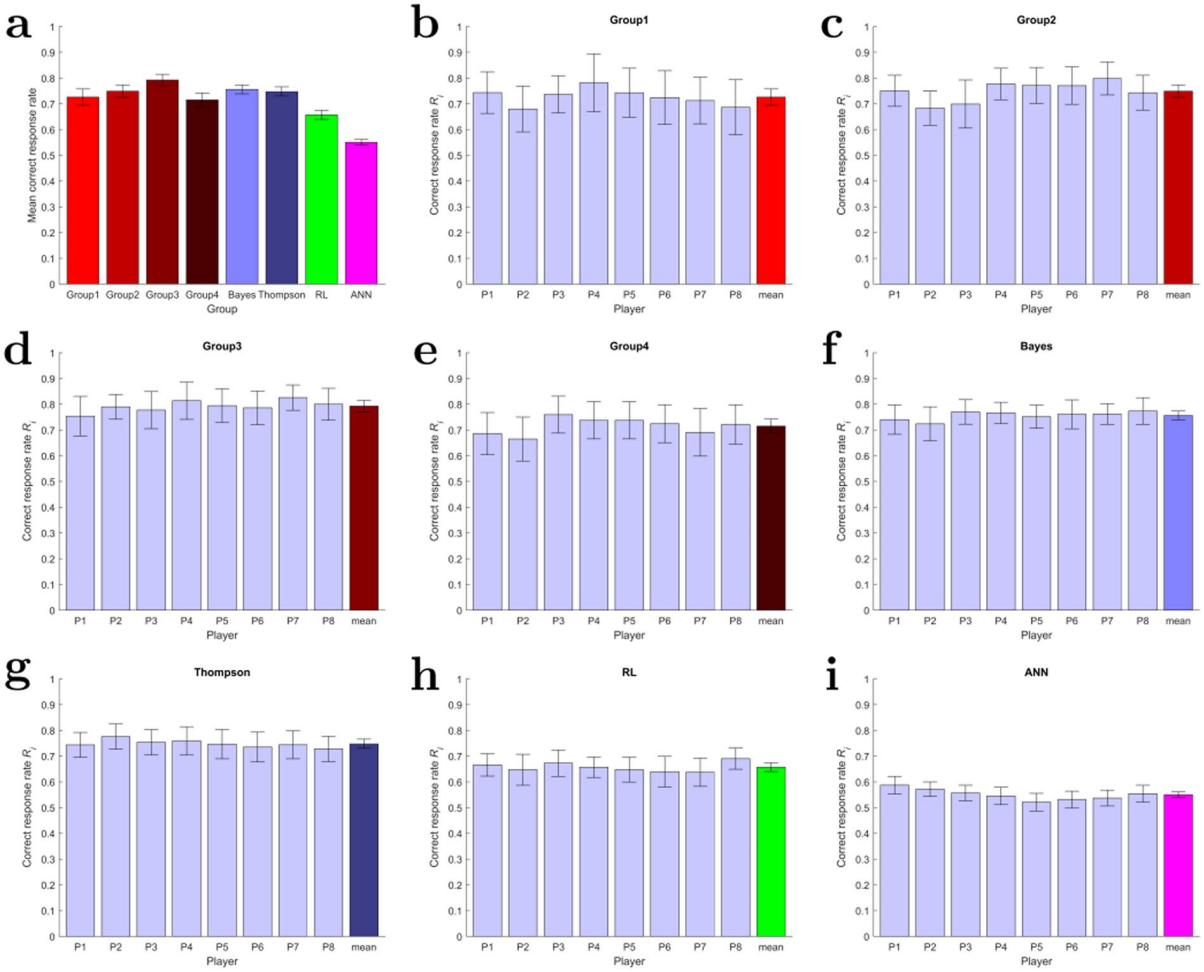
Individual correct response rates. (**a**) Average correct response rates in each group (**b**–**i**) Individual correct response rates. A single player’s action is deemed to be a correct response, if it had steered the cursor towards the target independent of the remaining group members. Error bars indicate 99% confidence intervals.

Despite the similarity between the best-fitting models and the human groups’ success rate, there are notable differences within every human group when it comes to individual correct response rates as displayed in Fig. 3. A single player’s action is deemed to be a correct response, if it had steered the cursor towards the target independent of the remaining group members. While we find a mean of about 75% of all individual decisions to be correct responses amongst the human players as well as in the Bayesian and Thompson simulated models, the human groups are slightly more heterogeneous in their response profiles. The simulated groups are homogeneous and mostly consist of copies of the average player, whereas the human groups include a range of good, average and bad players. Surprisingly this inhomogeneity within the groups of players hardly affects the overall success rate of the different groups that are very similar to each other with performance differences less than 5%. The simulated groups in contrast consist only of average units with much less variation in skill level of the involved players, which is why we can only compare the artificial units’ behavior to the mean performance of the human players. The RL and ANN models do not only have lower group success rates, but also diminished individual performances achieving correct responses around 66% and 55%, respectively (cf. Fig. 3). Remarkably, for all groups there is a significant gap between the average individual correct response rate and the overall group success rate, suggesting some kind of group synergy. For all but the ANN group the proportion of successful group decisions is approximately 10% higher than the proportion of individual correct responses. The worst model to approximate the human groups’ data is the ANN model with a gap of 5%.

#### Event synchronization

**Figure 4:**
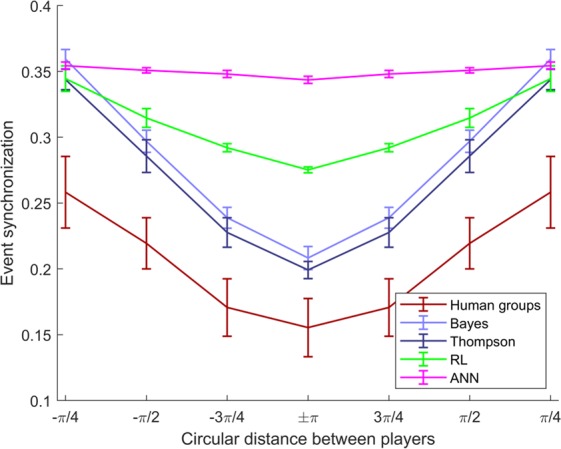
Event synchronization. Average event synchronization across all pairs of players as a function of the circular distance of the actual movement direction between players. Error bars indicate 99% confidence intervals.

In order to investigate possible explanations for the group synergy, we have to scrutinize the interactions between players. Since we know which players have similar movement directions, we know which players should be active together and which ones should not. We can therefore formulate the hypothesis that players that are separated by larger angular distances in movement direction, should co-activate less often. To measure players’ coactivation we utilize the measure of *event synchronization*, which has been previously proposed as a measure of similarity between two spike trains^31^. Intuitively, this measure defines a temporal neighborhood based on the inter-spike intervals around every spike of one time series and measures how often this neighborhood includes spikes of the other time series—see section Data analysis for details. The hypothesis therefore is that players with similar movement direction should be prone to produce more synchronous time series, with minimum synchronization for players that are opposite with circular distance $\pm \pi$. Figure 4 shows the event synchronization averaged across all pairs of human players and for all pairs of simulated players. As suggested by the hypothesis, we find that pairs of human players have higher event synchronization when they are closer together, and that the lowest event synchronization is recorded for pairs of players that are opposite. The *flatness* or depth of the resulting U-shape provides information about the level of cooperativity. This U-shaped pattern is reproduced by the Bayesian models and to a lesser extent by the RL model, but not by the ANN model. Generally, all the models have a higher event synchronization than the human players.

#### Action time correlation

**Figure 5:**
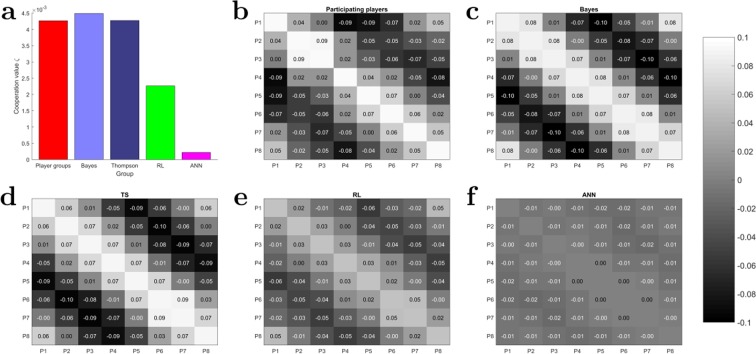
Action time correlation. (**a**) Cooperation values of all groups computed by the covariance between players action time correlation and success probabilities. (**b**) Action time correlation between all pairs of human players. (**c**–**f**) Action time correlation between all pairs of simulated players for the different models. For a level of significance $\alpha =0.05$, we find that all correlations are significantly different from zero.

Another measure indicative of the synergy between players’ interaction can be constructed from the action time correlation. The action time correlation between two actors is measured by the Spearman correlation between the two binary vectors that represent whether the actors have been active or inactive at a discrete point in time (see Supplementary Table S1). As there are eight actors, there are 64 combinations of pairs that can be arranged in a matrix, where the diagonal represents the degenerate case of perfect correlation between actors that are paired with themselves. Naturally, this matrix is symmetric, as there are only 28 distinct pairs in total. To improve readability in Fig. 5, the players are arranged in the matrix in a way that reflects their neighborhood relationships. Accordingly, we would expect the highest correlation close to the diagonal and the two off-diagonal corners (due to circular geometry), and lower correlations in between, because players that are closer together should be more likely to act together than players with opposing movement directions.

Subjects’ action time correlations can be seen in Fig. 5b averaged over all groups, the action time correlations of the four different models are shown in Fig. 5c–f. It can be clearly seen that subjects’ correlations exhibit the intuited neighborhood relationships as described, and that this pattern is qualitatively mimicked by all models. However, quantitatively, the observed absolute values of action time correlation are only matched by the Thompson model and the Bayes model, whereas the reinforcement learning agents and the ANN units have a reduced amplitude modulation of the correlation. Especially, the ANN model exhibits hardly any variation.

To reduce the groups’ cooperativity to a scalar value, we construe a measure of cooperation for a group of binary actors by relating the correlation between players to the success probability of joint actions. In our task, the success probability of an interaction between two players is defined by the number of instances where the two players were active simultaneously and the cursor’s distance to the target was decreased compared to the number of all instances where the two players were active simultaneously irrespective of what the rest of the players were doing. The central idea is that when players tend to act together with high correlation, interactions that involve the two players should also imply a high success probability for the group and vice versa. Such useful correlations should lead to a positive measure of cooperation. If players act independently and randomly, the measure of cooperation should be zero. If players act antagonistically, the measure of cooperation should be negative. To obtain such a cooperation measure for the group, we determine the similarity between the correlation matrix and the success probability matrix between all pairs of players, where the similarity is computed through the covariance of all relevant matrix elements of the correlation and success probability matrix—see section Quantifying cooperation in Groups for details.

Comparing the cooperation measure for all groups in Fig. 5a, we find an average value of 0.0043 for the groups of participating players, 0.0045 for the Bayesian model, 0.0043 for the Thompson model, 0.0023 for the RL model and 0.0002 for the ANN model. This implies a similar degree of cooperation within the groups of human players and the Bayesian and especially the Thompson simulation and to a lesser extend within the RL model. In contrast, the ANN agents produce spike trains that contain hardly any information about the units’ neighborhood relations, implying mostly randomly formed active player sets for every time step.

### Quantifying Group Learning

In order to quantify the learning process of the different groups and the individual players, we analyse the temporal evolution of success rates and the temporal variation of the spike-triggered average (STA), that is the circular mean of target difference vector angles that directly precede the decision-maker’s response. The STA is a standard tool in neurophysiology to characterize response properties of neurons and we use it here to describe changes in the receptive field of the neuron-like decision makers.

#### Improvements in success rates

**Figure 6:**
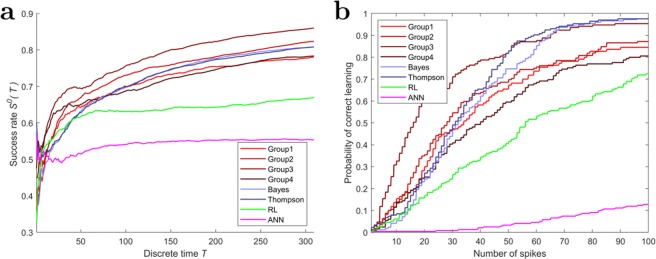
Time-dependent success rates and learning duration. a Success rate as a function of time for the four human groups and the four computational models. (**b**) Proportion of batches with learning durations averaged across all decision-makers for all groups.

Figure 6a depicts the success rate averaged across all batches for each group of decision-makers at each point in time. The curves therefore show how the groups success rate improved over ten consecutive trials with the same assignment of movement directions. At the beginning of the batch all groups start at a 0.5 chance level and then the human and Bayesian models progress up to approximately 80%. The RL model is slower and achieves only $\sim \mathrm{70 \% }$. The ANN model is even slower and improves only slightly up to 60% after 3500 time steps, as it takes much longer to reach the target than the other models. While the Bayesian models match human learning progress quite well, there are two notable differences. Initially, the human groups’ improvements are steeper than any of the models and then level off. Moreover, the variance across batches is significantly lower in the Bayesian models, due to the fact that the simulated groups consist of homogeneous units (see Supplementary Fig. S1).

#### Changes in the spike triggered average

**Figure 7:**
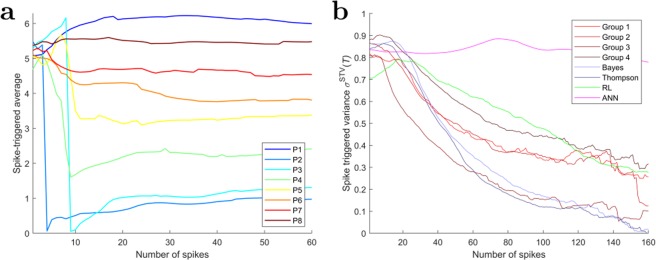
Changes in spike triggered average across time. (**a**) Temporal evolution of the mean target difference vector angle preceding a group response for each decision-maker in group 3. (**b**) Time dependency of the average spike triggered variance. The abscissa displays the ordinal spike number for each player. The estimate becomes more variable for large number of spikes due to the fact that most batches are terminated before reaching 100 spikes for many players.

In general, the spike-triggered average (STA) is the average stimulus preceding a neural response. In our case, this corresponds to the cursor/target configuration that triggered a response on the part of the decision-makers. As we can measure the cursor/target configuration by the scalar quantity of the target difference vector angle, the STA simply requires the computation of a circular mean for each decision-maker. Figure 7a shows for an exemplary group how the STAs of all eight players change over the time frame of ten consecutive trials, averaged across all batches. The initial estimate for all players starts at a random location due to the ill-definedness of the circular mean of uniformly distributed samples. Over time the estimates differentiate for each player to approximate the assigned displacement direction at the end of learning. Over the course of this adaptation process, the variance in the stimuli that trigger players’ responses decreases, as can be seen in the spike-triggered variance depicted in Fig. 7b, which is a clear indication that decision-makers’ responses become more and more specific. Ultimately, the spike triggered variance decreases for all groups to values below $0.1\,ra{d}^{2}$. The most rapid decline can be observed for the third experimental group, and the Bayes and Thompson models. The first, second and fourth experimental group as well as the RL group undercut this boundary more slowly and the ANN group is the slowest that reaches the boundary after  more than 500 steps.

The spike-triggered variance only measures the precision, but not the accuracy of learning. To measure accuracy, we introduce the learning duration ${T}_{i}^{\ast }$ for player $i$ as the point in time from which onwards the STA remains in a broad neighborhood of the correct movement direction—see Methods for details. Once the learning duration is completed, the decision-maker contributes to a movement towards the target whenever the stimulus that triggered the response is close to the current STA. Figure 6b shows for all possible spike counts the proportion of batches with learning durations less or equal the spike count averaged across all decision-makers. Naturally, the longer the duration, the higher the chance that decision-makers have converged already. We fitted the curves with cumulative exponential distributions using a single rate parameter. The human groups and the RL model fit the exponential curve rather well, whereas the Bayesian models and the ANN model are initially slower to converge than the humans, but then converge rather rapidly (see Supplementary Fig. S2 for details). The human groups and the Bayesian models achieve 70% correct learning after approximately 50 spikes, whereas the RL model requires $\sim 100$ steps and the ANN model $\sim 400$.

**Figure 8:**
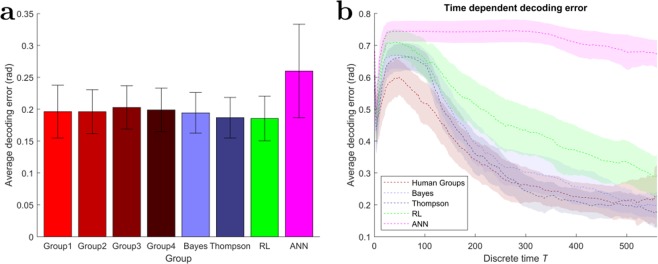
Decoding error. (**a**) The average decoding error produced by the different groups of players calculated with the spike triggered average of the whole batch. Error bars indicate 99% confidence intervals. (**b**) The temporal evolution of the decoding error displayed for the average human group and all four simulated groups. The shaded areas indicate 99% confidence intervals.

In order to not only assess the average individual learning progress but also the learning progress of the groups as a whole, we gauge the ability of the group to represent stimuli in the form of target difference vector angles in terms of an eight-dimensional spiking activity. To this end, we consider the groups’ response as a neural encoding of the stimulus given by the target difference vector angle. In particular, we assume a population vector encoding where each decision-maker’s preferred direction is given by the STA. Abstractly, we study how well a stimulus point can be reconstructed from coordinates in an overcomplete coordinate system where each axis is given by the STA of a particular decision-maker. We can then calculate a population vector encoding of every stimulus encountered by the group and compare it to the actual target difference vector. The average radial error between the angle of the group’s population vector and the actual target difference vector angle measures the learning progress of the group in terms of representing the target difference vector angles—see section Quantifying learning process for details. Figure 8b shows the temporal evolution of the decoding error, where the Bayesian models and the human groups are characterized by a similar temporal profile. Figure 8a shows how all the models except the ANN model eventually converge and have similar decoding errors of approximately $\mathrm{0.18(}\approx {10}^{\circ })$ error, whereas the ANN error remains around approximately 0.25.

## Discussion

In this study we investigate a novel experimental paradigm, in which a group of humans is joined together to solve a common sensorimotor task. Every player takes the role of a binary decision-maker that reacts to a common visual input. We quantify to what degree these individual neuron-like players behave as a single unit, analyze their learning progress and cooperative performance, and compare the observed human group behavior to four different computational models. In both the models and the experimental data, we found that group performance exceeds the average individual performances, indicating a certain degree of synergism and cooperation benefit. Moreover, we found that the best-fitting models are those where each individual decision-maker maintains an internal model about the consequences of their binary action. Assuming a Bayesian formalism, these internal models could cope well with the uncertainty of the triggered consequences and they excelled in predicting the human players’ performance, success rates, individual correct response rates, action time correlations, and the same modulation of event synchronization as the human groups. In contrast, model-free learning methods based on rewards, that signal an increase or decrease of distance to the target, were not able to reproduce the features characterizing the human players, even though both the reinforcement learner and the perceptron learner are still capable of solving the game. Both these models are much slower learners compared to the model-based Bayesian learners, because they process less feedback information and struggle to cope with stochastic rewards. Especially the perceptron model fails to explain almost every single measure that we studied.

In addition to the steady-state behavior observed towards the end of our trial batches, we studied the adaptation process of the different groups when exposed to novel and unknown displacement directions at the beginning of each trial batch. We found that the temporal evolution of the success rate and the spike-triggered average stimulus indicate that the learning processes of human players and the Bayesian model units exhibit similar dynamics with comparable learning durations. Furthermore, the variance of the spike-triggered average indicates that the receptive field of most human players narrows significantly within their first 100 spikes, which is reproduced by all models except the perceptron model. Overall, our results suggest that group coordination in a multi-player sensorimotor task is best explained by individual players that maintain an internal model about the consequences of their actions.

Coordination between multiple actors in sensorimotor tasks has so far been mostly studied in dyads, i.e. interactions between two players. Research on joint action has focused on shared representations between actors, shared attention, the mechanisms to predict actions and to integrate these predictions into the interaction^32^. These previous studies have found, for example, that reducing variability is used as a coordination strategy to achieve predictability^33^. In general, the ability to perform complementary actions in joint tasks and to imitate other actors is particularly advanced in humans, and is developed early in childhood. In line with our findings, a large body of evidence suggests that pairs of humans involved in joint action have internal models and expectations about the consequences of their own actions and about the actions undertaken by their interaction partner^33–39^. Quantitative models of sensorimotor interactions have so far also focused on the two-player scenario within a game-theoretic setup^18,19,40^. In these games, pairs of human subjects were haptically coupled through force-fields generated by manipulanda, where the force-fields could change dynamically with the action of each player, reflecting the payoffs in a non-cooperative game. Interestingly, it was found that players’ sensorimotor interaction was well captured by game-theoretic Nash solutions, for example, in the Prisoners’ Dilemma, in contrast to cognitive versions of the same game. Sensorimotor interactions between more than two players have been investigated in the context of social loafing, where individual participants exert less effort in a group than when they act by themselves, for example in a game of tug-of-war^22^. In principle, interactions in our NeuronGame could be analyzed within the framework of cooperative game theory, where the main prediction would be that the optimal organization is a grand coalition of all players where at each instant of time those players are active that can move the cursor towards the target. In order to obtain non-trivial predictions, the experimental paradigm would have to be modified, but this was not the focus of our study.

Bayesian models in sensorimotor interactions have been previously investigated in the context of communicative sensorimotor behavior^20^. Theoretically, such interactions can be considered as a signalling game where one player forms a Bayesian belief about the true type of the other player that is only communicated indirectly through a noisy signal. Since human behavior was well captured by this Bayesian model, this corroborates our conclusion that Bayesian inference may provide a useful framework in the context of understanding group behavior. There is already ample evidence that Bayesian integration and inference is a powerful modelling tool for individual human learning and motor control in uncertain environments as representing uncertain knowledge enables actors to efficiently use sensory information during action^41–44^. Bayesian models have also been very successful in explaining perceptual processes and illusions building on the Helmholtzian notion that perception is a model-based inference process^45,46^. Our results suggest that the same kind of principles that govern individual motor control and learning may also underlie group interaction and cooperation^47^.

The question of how cooperation could arise during the course of evolution has been an intense field of study both in evolutionary biology and theoretical biology^1,48–50^. Mathematical principles for the evolution of cooperation have been extensively studied over the last decade both with respect to the molecular level and to describe interactions between complex organisms^48,49^. Cooperation is typically divided into mutualism and altruism, where interactions of the first kind are beneficial to the individual as well as the group, in contrast to the second kind, where an individual seemingly sacrifices its own well-being for the sake of others. Explanations of cooperative behavior include genetic arguments (e.g. inclusive fitness of helping relatives), behavioral arguments (e.g. reciprocity, tit-for-tat), and models of group selection^50,51^ suggest five rules for the evolution of cooperation between individuals, including kin selection^52^, direct and indirect reciprocity e.g.^53^, group selection^54^ and network reciprocity^55^. On an abstract level we may ask about the principles that govern cooperation across systems that we may regard as consisting of multiple decision units^56^. This may involve research questions on group learning^57^, individual versus group decision-making^58^, and cooperation within humans, animals and even cellular organisms^3,4,53,59^. From this perspective, our results and methods to measure cooperation in a group of humans in a sensorimotor task may also be of interest to the broader research on cooperation in groups of animals^60,61^.

In our experiment each individual player was acting as a neuron-like decision-maker with a binary choice generating a spike train that together with the spike trains of the other players would generate behavior. We could therefore think of our group of neuron-like decision-makers as a little “brain”. In this abstract model, the “brain” is considered as an economy of little interacting agents that together learn to control an unknown environment. We could therefore ask to what extent this analogy may tell us something about neural processing in real brains. First of all, the ensemble of decision-makers differs from neural ensembles in a real brain in that the neuron-like decision-makers only communicate through the shared visual input, whereas real neurons directly communicate with each other through their synapses. Nevertheless, the human decision-makers might resemble real neurons on a more abstract level in that they create a model of their input and behave according to this model, which has previously also been proposed for neurons^62^. In this vein, it is argued that short-term changes in synaptic efficacy may be interpreted as implementing an optimal estimator of the presynaptic somatic membrane potential. Thus, human decision-makers in our experiment appear to estimate hidden states in their environment (e.g. their movement direction) in analogy to a biological neurons’ synapses that estimate the hidden state of the presynaptic neuron (e.g. presynaptic membrane potential) which could be represented by the local postsynaptic potential at an excitatory synapse^62–65^. Moreover, neurons are known to change their spiking responses to relevant signals with training^66^. A similar receptive field plasticity is reflected in our experiment as participants are initially unaware of their displacement direction and learn to correctly react to their environment. Therefore the size of the receptive field of the decision-makers diminishes over time and adapts dynamically to the environment. In summary, both, human decision-makers and neurons are able to process incoming signals, respond to information provided by the environment, and adapt their behavior and their representation of internal states.

## Conclusion

**Figure 9:**
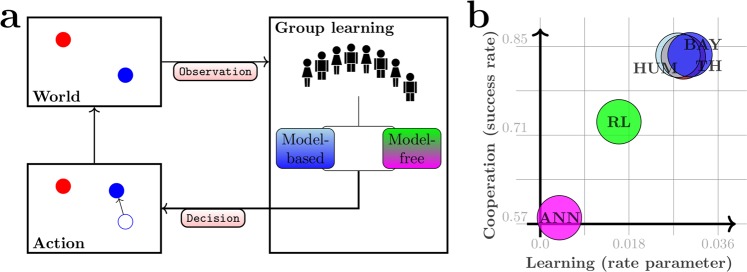
Results summary. a An overview of the key elements of the game. At every point in time the position of the target and the position of the cursor provoke a group decision that results in a cursor displacement on the screen. This movement is observed by the group and members of the group update their knowledge about the effect of their decisions and change their behaviour accordingly. To describe the human learning we compare four different models that can be categorized as either model-based (Bayes and Thompson) or model-free (Q-learning, Perceptron). (**b**) Sketch of the performance in terms of learning progress and cooperation of human learners and the four different models. The abscissa shows the rate parameter of an exponential distribution whose cumulative distribution function was fitted to the learning duration data shown in Fig. 6b and the ordinate shows the success rate as shown in Fig. 2a.

In this study we present an experimental paradigm to investigate sensorimotor coordination in a group of humans that act as neuron-like binary decision units. Our results are summarized in Fig. 9. We compare human group learning against four different learning models that can be classified as model-free and model-based—see Fig. 9a. In the latter case the decision-makers explicitly learn to predict the consequences of their actions, whereas in the former case only the utility of the action is considered in the learning process. Figure 9b shows the performance of the presented models compared to the human participants. Even though all models are able to solve the game in principle, the model-free reinforcement learning and perceptron models fail to capture human behavior in terms of learning and cooperation measures. In contrast, the Bayesian model-based simulations learn as fast as the human players and their cooperation measures match closely with the results of the participating human players. In summary, we find evidence that human behavior in a sensorimotor group learning task is best explained by assuming that each decision-maker forms an internal model over their environment.

## Methods

### Experimental methods

#### Participants

Thirty-two (nine female and twenty-three male) students at Ulm Univeristy participated in the experiment. All participants were naïve with respect to the purpose of the study. The experiment lasted over the course of two hours. Participants were compensated for their time with 10 per hour.

#### Ethics statement

All participants gave informed consent and the study was approved by the ethics committee of Ulm University. All experiments and methods were performed in accordance with the relevant guidelines and regulations.

#### Setup

In the experiment eight subjects sat in front of a projector screen (2.09 *m* times 1.14 *m*, resolution 1980 × 1080 pixels) to play the NeuronGame, an applied computer game adapted from an e-learning software that was mainly developed for teaching purposes at the Bernstein Center of the Albert Ludwigs University of Freiburg. Each participant held a push-button controlled by an Arduino Leonardo Microcontroller. The participants were placed in a row of two arranged as a half circle and they were told to hide their buttons from the views of the other players. In order to prevent auditory cues from clicking sounds produced by the other players, subjects wore headphones or earplugs, while a soundtrack of rain sounds and white noise was played on a speaker placed behind the participants. This way it was ensured that the only cue for the effect of each player’s action was the visual response on the screen.

#### Experimental design

Each trial in the NeuronGame consists of a group of eight players steering a cursor from a start position to a target without any obstacles being present—see Fig. 1 for an illustration. Every player has to decide at every point in time whether or not to press their button. Each player *i* is assigned an unknown movement direction angle ${\mu }_{i}\in \mathrm{[0,}\,2\pi )$, such that pressing the button leads to a small displacement (approx. length of 10 pixels) of the cursor into that direction. The movement directions were randomized every ten trials, where each player is randomly assigned one of eight equiangular directions of a rotated wind rose. In contrast, target and start position were randomized every trial by uniformly sampling a start position and an angle, such that the target position was determined by that angle with respect to the start position and by a fixed distance of 840 pixel lengths. Samples close to the edge of the screen (within a 10% brim) were rejected. At the end of every successful trial, the cursor’s trajectory and the action times (in milliseconds) of all players were saved and the game was paused for a visible three second countdown before the next trial started. Every ten trials when movement directions were randomized another three second countdown was displayed accompanied by a flashing orange screen and the note that the directions were being randomized. In total, the game was partitioned into 8 sets of 5 batches consisting of 10 trials each, meaning that each ensemble had to play 400 trials successfully, except the first group that terminated early after 5 sets.

### Data analysis

#### Time discretization

For purposes of data analysis, we discretize time in the experiment by finding the largest time interval that separates any two consecutive spikes of each single player. In the following we refer to events happening within this time interval of approximately 130 ms, as occurring at the *same* point in (discrete) time. This way we can ensure that every player was never active more than once at each time point. Supplementary Table S1 shows the time intervals’ length in milliseconds chosen for the four different groups of participating players.

#### Notation

In order to measure the different groups’ performance we propose in the following several measures and methods of measurement to assess the level of group learning and cooperation and outline their relevance to the analysis. Every group of simulated agents or human players consists of eight decision-makers that repeatedly decide whether to press their button or stay idle at every point in time ${t}_{1},\ldots ,{t}_{{\rm{end}}}$. By playing the NeuronGame they generate data in the form of sequences of group actions ${\bf{A}}={({{\bf{a}}}^{(t)}\in {\mathrm{\{0,}\mathrm{1\}}}^{8})}_{t\in \mathrm{\{1,}\ldots ,N\}}$ representing button presses that can be stored in a matrix $A\in {{\mathbb{R}}}^{N\times 8}$ where *N* is the number of time points. The actions in turn generate cursor trajectories ${\bf{S}}={({{s}}_{t}\in {{\mathbb{R}}}^{2})}_{t\in \mathrm{\{1,}\ldots ,N\}}$ representing the path from the start to the target area, which again can be represented by a matrix ${\bf{S}}\in {{\mathbb{R}}}^{N\times 2}$. Moreover, for the analysis we keep track of the average displacement experienced by each player *i* for all the time points when they were active up to the current moment *t*_*j*_.

#### Success rates and correct response rates

In order to evaluate players’ performance, we measure the success rate ${S}^{\delta }(T)$ for group actions *a* where at least one player was responding. In particular, such actions are considered successful, if they decrease the cursor’s distance to the target at least by a minimum distance of $\delta  > 0$. We define the time dependent success rate at time $T < N$ of a batch of trials as the percentage of successful time points up until time *T*, namely

1 $${S}^{\delta }(T)=\frac{1}{T}\mathop{\sum }\limits_{t=1}^{T}H(\Vert \tau -{s}_{t}\Vert -(\Vert \tau -{s}_{t+1}\Vert +\delta ))$$

Moreover, Eq. (1) allows defining player-specific success rates ${S}_{G}^{\delta }(T)$, where *G* denotes a subset of players that have to be active at all the time points that are considered. For example, we can define the pairwise success rate for a pair of players *i* and *j* as

$${S}_{i,j}^{\delta }(T)=\frac{\mathop{\sum }\limits_{t=1}^{T}{{\bf{A}}}_{i,t}{{\bf{A}}}_{j,t}H(\Vert \tau -{s}_{t}\Vert -(\Vert \tau -{s}_{t+1}\Vert +\delta ))}{\mathop{\sum }\limits_{t=1}^{T}{{\bf{A}}}_{i,t}{{\bf{A}}}_{j,t}}$$

for all the time points where both player *i* and *j* contributed to the action.

Independent of actual success, we call an individual decision a correct response whenever the player’s action would have decreased the distance to the target if the player had acted by itself. While many times a correct response may be accompanied by a success, other times the correct response of a single player may be overridden by a group of individuals producing incorrect responses. We define the correct response rate *R*_*i*_ of individual *i* up to time $T < N$ as

$${R}_{i}(T)=\frac{\mathop{\sum }\limits_{t\mathrm{=1}}^{T}{{\bf{A}}}_{i,t}{H}(\Vert \tau -{{s}}_{t}\Vert -\Vert \tau -{{s}}_{i}^{\ast }\Vert )}{\mathop{\sum }\limits_{t\mathrm{=1}}^{T}{{\bf{A}}}_{i,t}},$$

where ${s}_{i}^{\ast }$ corresponds to the cursor position that would have been achieved if the movement direction was solely determined by player *i*.

### Event synchronization

To measure similarity between two players’ response profiles, we utilize *event synchronization*, which has been previously proposed as a measure of similarity between two spike trains^31^. Intuitively, this measure defines a temporal neighborhood based on the inter-spike intervals around every spike of one time series and measures how often this neighborhood includes spikes of the other time series. More formally, let ${T}^{i}=\{{t}_{1}^{i},\ldots ,{t}_{{m}_{i}}^{i}\}\,:\,=\{t|{{\bf{A}}}_{i,t}=1\}$ be the points in time when player *i* is active. Then define the temporal neighborhoods $\tau$ and counting functions *J* for players *i* and *j* according to:

2 $${\tau }_{k,l}\,:\,=\,\frac{1}{2}\,min\{{t}_{k+1}^{i}-{t}_{k}^{i},{t}_{k}^{i}-{t}_{k-1}^{i},{t}_{l+1}^{j}-{t}_{l}^{j},{t}_{l}^{j}-{t}_{l-1}^{j}\}\,\,\,{\rm{a}}{\rm{n}}{\rm{d}},$$

3 $${J}_{k,l}\,:\,=\{\begin{array}{cc}1 & {\rm{i}}{\rm{f}}\,0 < {t}_{k}^{i}-{t}_{l}^{j}\le {\tau }_{k,l}\\ \frac{1}{2} & {\rm{i}}{\rm{f}}\,{t}_{k}^{i}={t}_{l}^{j}\\ 0 & {\rm{o}}{\rm{t}}{\rm{h}}{\rm{e}}{\rm{r}}{\rm{w}}{\rm{i}}{\rm{s}}{\rm{e}}\end{array}$$

such that we can define the measure of synchronicity between player *i* and player *j* as

4 $${{\rm{ES}}}_{i,j}\,:\,=\frac{\mathop{\sum }\limits_{k=1}^{{m}_{i}}\mathop{\sum }\limits_{l=1}^{{m}_{j}}({J}_{k,l}+{J}_{l,k})}{\sqrt{{m}_{i}{m}_{j}}}$$

The idea is that subjects with similar movement direction should be prone to produce more synchronous time series. Hence, if we denote the circular distance between players’ movement directions by $d\in \left\{-\frac{\pi }{4},\,-\,\frac{\pi }{2},\,-\,\frac{3\pi }{4},\,\pm \,\pi ,\frac{3\pi }{4},\frac{\pi }{2},\frac{\pi }{4}\right\}$ we would expect the lowest average synchronization for opposing players with $d=\pm \,\pi$ with average synchronization increasing for $|d| < \pi$, resulting in a U-shaped relationship. The *flatness* or depth of the resulting U-shape provides information about the level of cooperativity.

### Action time correlation

In order to capture the groups’ ability to work together as a single unit, we determine to what extent pairs of players that are active together also contribute positively to the group performance on average. To this end, we compute players’ action time correlation and put it in relation to players’ success rates. Formally, the action time correlation between two decision-makers *i* and *j* is measured by

5 $${C}_{i,j}=\frac{\mathop{\sum }\limits_{t=1}^{N}({{\bf{A}}}_{i,t}-{{\bf{A}}}_{i})({{\bf{A}}}_{j,t}-{{\bf{A}}}_{j})}{({\bf{N}}-\mathrm{1)}{{s}}_{{{\bf{A}}}_{i}}{{s}}_{{{\bf{A}}}_{j}}},$$

where **A**_*i*_ and **A**_*j*_ are binary vectors that indicate whether the actors pressed their button or stayed idle at a particular point in time, ${N}\,:\,=|\{{t}_{1},\ldots ,{t}_{{\rm{end}}}\}|$ indicates the time steps, and **A**_*i*_ represents the sample mean across time and ${s}_{{{\bf{A}}}_{i}}$ the sample standard deviation of **A**_*i*_.

Following a similar hypothesis as above, namely that spike trains of subjects with similar movement direction are prone to be correlated, we define the cooperation measure $\zeta$ as the covariance between the action time correlation and the probability of successful cooperation between players. The central idea of the cooperation measure $\zeta$ is that for pairs of players with a high action time correlation, the interactions that involve the two players should also imply a high success probability for the group and vice versa. Since there are ${N}_{p}=\frac{\mathrm{8(8}-\mathrm{1)}}{2}=28$ unique combinations of different pairs of players, we measure the linear covariation between corresponding entries in the correlation and in the success probability matrix by calculating the sample covariance. With vectors C and S that contain all *N*_*p*_ entries of the strict upper diagonal matrices of ${({C}_{i,j})}_{1\le i,j\le 8}$ and ${({S}_{i,j})}_{1\le i,j\le 8}$, respectively, the cooperation measure within the group is then defined as

6 $$\zeta \,:=\frac{1}{{N}_{p}-1}\mathop{\sum }\limits_{k\mathrm{=1}}^{{N}_{p}}({{C}}_{k}-\bar{{C}})({{S}}_{k}-\bar{{S}})$$

where $\bar{{C}}$ and $\bar{{S}}$ denote the sample mean across the *N*_*p*_ entries. Negative values of $\zeta$ correspond to a dysfunctional group of decision-makers where unsuccessful pairs of players are likely to be active at the same time and positive values of $\zeta$ indicate cooperation where successful pairs of decision makers are more likely to spike at the same time.

### Quantifying learning progress

To assess decision-makers’ consistency in their responses we can determine the average stimulus that is driving their behavior whenever they are active. In neurophysiology such an average stimulus is known as a spike-triggered average (STA) and can be used to determine the receptive field of a nerve cell^67^. In our experiment the stimulus is given by the target difference vector angle $\phi (\tau -{{s}}_{t{\prime} })$ seen by all decision-makers. Accordingly, we can determine the spike-triggered average

7 $${\phi }_{i}^{STA}(t)={\rm{atan}}2\left(\frac{1}{{m}_{i}(t)}\mathop{\sum }\limits_{t{\prime} =1}^{t}{{\bf{A}}}_{i,t{\prime} }\,\sin (\phi (\tau -{{s}}_{t{\prime} })),\frac{1}{{m}_{i}(t)}\mathop{\sum }\limits_{t{\prime} =1}^{t}{{\bf{A}}}_{i,t{\prime} }\,\cos (\phi (\tau -{{s}}_{t{\prime} }))\right)$$

where ${m}_{i}(t)={\sum }_{t{\prime} \mathrm{=1}}^{t}{{\bf{A}}}_{i,t{\prime} }$ is the number of times player *i* has triggered a spike until time *t*. Similarly, we can ask for the variance of the set of stimuli that triggered a response, i.e. a spike triggered variance (STV), which in our case is given by

8 $${\sigma }_{i}^{STV}(t)=1-\sqrt{{\left(\frac{1}{{m}_{i}(t)}\mathop{\sum }\limits_{t{\prime} =1}^{t}{{\bf{A}}}_{i,t{\prime} }\sin (\phi (\tau -{{s}}_{t{\prime} }))\right)}^{2}+{\left(\frac{1}{{m}_{i}(t)}\mathop{\sum }\limits_{t{\prime} \mathrm{=1}}^{t}{{\bf{A}}}_{i,t{\prime} }\cos (\phi (\tau -{{s}}_{t{\prime} }))\right)}^{2}}$$

with the idea being that for learning decision-makers the spike triggered variance should decrease over time as responses become more consistent. Moreover, we can define the *learning duration* for player _i_ as the first point in time ${T}_{i}^{\ast }$ where the spike-triggered average ${\phi }_{i}^{STA}$ remains in a broad neighborhood of the true movement direction angle ${\mu }_{i}$ in all future time points, such that $\forall t\ge {T}_{i}^{\ast }\,:\,{d}_{C}({\phi }_{i}^{STA}(t),{\mu }_{i})\le \frac{\pi }{2}$.

To assess not only individual learning but also progress of group learning we regard the group’s spiking activity as a neural population (see^68^ for other models for reading neuronal population codes) vector that encodes the current target difference vector angle. To this end, each decision unit at time is assigned a two-dimensional unit vector ${{e}}_{i}^{STA}$ representing the preferred stimulus ${\phi }_{i}^{STA}$, such that we can decode any stimulus ${{v}}_{t}=\tau -{{s}}_{t}$ by the population vector

9 $${{v}}^{{\rm{pop}}}(t)=\mathop{\sum }\limits_{i\mathrm{=1}}^{8}\langle {{v}}_{t},{{e}}_{i}^{{\rm{STA}}}\rangle {{e}}_{i}^{{\rm{STA}}}\mathrm{}.$$

The average decoding error

10 $$\varepsilon =\frac{1}{N}\mathop{\sum }\limits_{t=1}^{N}{d}_{C}({{v}}^{{\rm{pop}}}(t),{{v}}_{t})$$

can then be used as a measure of how well the group as a whole has learned to represent the target difference vector stimulus.

### Computational models

#### Model structure

**Figure 10:**
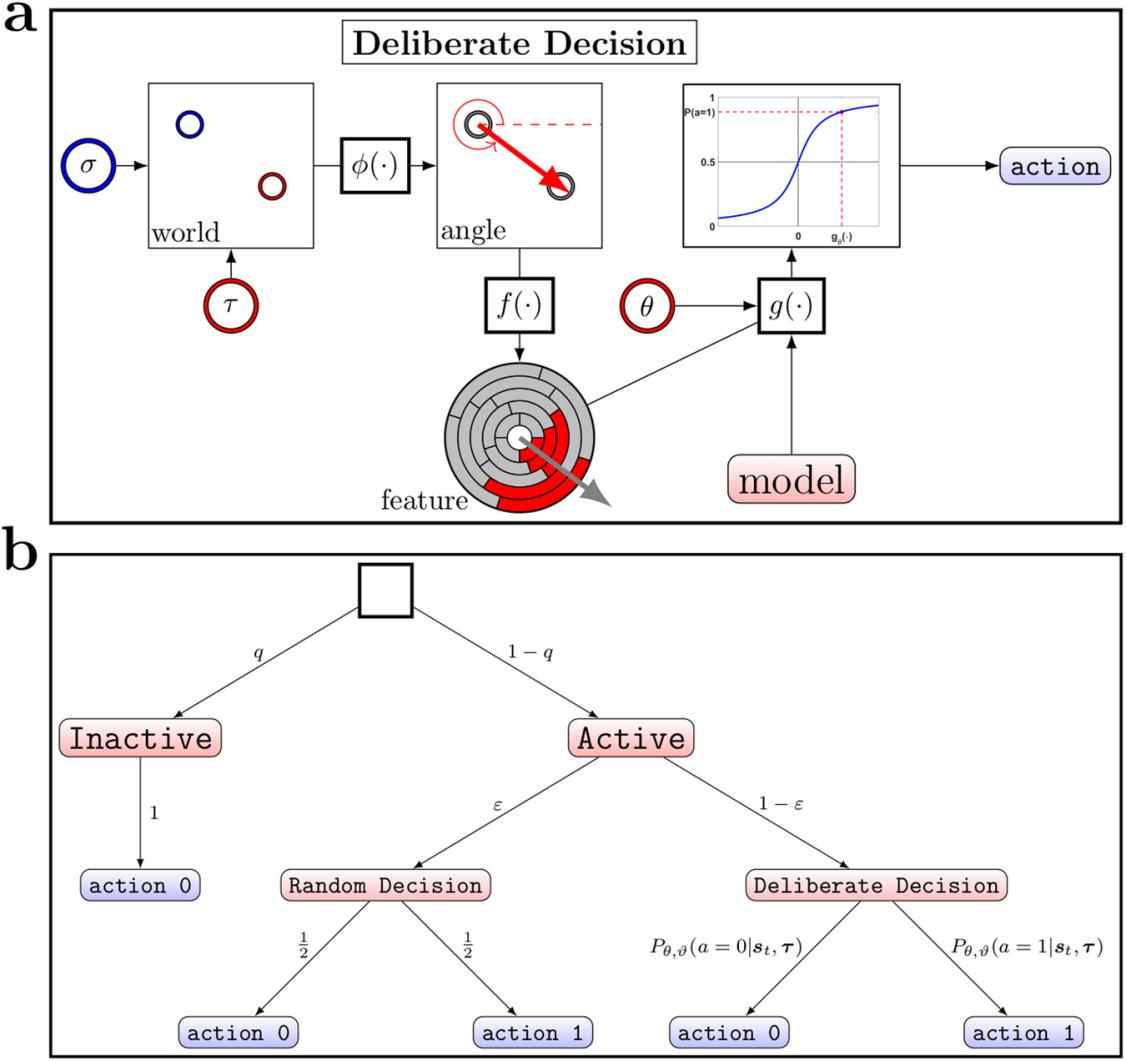
Decision-maker. Detailed sketch of the decision making process for the simulated units. (**a**) Overview of the deliberate decision, following a sigmoid decision function strategy. In a first step the world-state is determined. The function $\phi$ maps this state, i.e. the position of the cursor and the position of the target, onto the target difference vector angle. This angle is then transformed into a binary feature vector. (**f)** The activation level $g(\,\cdot \,)$ then determines a scalar value, i.e. a reaction to *f*, according to his type and his existing knowledge $\theta$. This value is then used in a sigmoidal decision function that determines the decision-maker’s action. (**b**) Schematic of the binary decision-maker’s structure. In a first step the decision-maker is either with probability *q* forced to staying idle (action 0) or he is allowed to follow an $\varepsilon$-greedy strategy with probability $1-q$. Following the $\varepsilon$-greedy strategy, the decision-maker chooses his action at random with a probability of $\varepsilon$ or sticks to an atan decision function with probability $1-\varepsilon$. The activation level ${g}_{\theta }(f)$ to the feature f used to evaluate the sigmoid decision-function is a scalar that is large for features similar to the best guess of the decision-maker’s movement direction and negative for features representing antagonistic movement directions.

All models consist of eight identical decision-making units whose parameters $\varTheta =({\theta }_{1},\ldots ,{\theta }_{8})$ are tuned over the course of a batch. Like in the real experiment, every simulated unit can decide whether to *press its button* or not, given the target position $\tau$ and the cursor position s_*t*_ at time_t_. Accordingly, the decision of unit *i* is denoted by ${a}_{i}\in \mathrm{\{0,1\}}$, such that the group of decision-makers generates a binary vector ${a}=({a}_{1},\ldots ,{a}_{8})$ of decisions at every point in time. In order to improve readability, we drop the index *i* in the remainder. As illustrated in Fig. 10, we assume the following processing pipeline for all decision-making models:

- First, the target difference vector $\tau -{{s}}_{t}$ is computed from target and cursor positions and then translated into a target difference vector angle $\psi (\tau -{{s}}_{t})$, where $\psi ({x})={\rm{atan}}\mathrm{2(}{x}_{2},{x}_{1})\,\mathrm{mod}\,2\pi$ is an angle in the interval $\mathrm{[0,}\,2\pi )$ and *x* is the vector consisting of the scalar components $({x}_{1},{x}_{2})$. In order to consider effects of observation noise, we assume that the target difference vector angle is contaminated by Gaussian noise, with representation $\phi (\tau -{s})=(\psi (\tau -{s})+\eta )\mathrm{mod}\,2\pi$ where $\eta \sim {\mathscr{N}}\mathrm{(0,}\,{\beta }_{3})$. The observation noise variance ${\beta }_{3}$ is a component of the hyper-parameters ϑ that are kept fix over the entire simulation.
- Second, the noisy target difference vector angle is mapped into a binary feature vector ${f}(\phi )$ that can be thought of as a basis function representation with rectangular basis functions. The feature vector $f(\phi )=({f}_{1},\ldots ,{f}_{nm}{)}^{{\rm{\top }}}$ is constructed from *m* tilings, each consisting of *n* divisions called *tiles*. If the value of $\phi$ falls within a tile the feature value is one, otherwise zero:

$${f}_{(i-1)n+j}=\{\begin{array}{cc}1 & {\rm{i}}{\rm{f}}\,\,i-1\le \frac{{\rm{n}}{\rm{m}}\phi -2\pi j}{2\pi m} < i\\ 0 & {\rm{o}}{\rm{t}}{\rm{h}}{\rm{e}}{\rm{r}}{\rm{w}}{\rm{i}}{\rm{s}}{\rm{e}}.\end{array}$$

Therefore, each block of *n* entries of *f* has exactly a single one and $n-1$ zeros, that is each vector *f* has *m* ones and *m*(*n* − 1) zeros. In total, there are *nm* different feature vectors $\{{{f}}^{\mathrm{(1)}},\ldots ,{{f}}^{(nm)}\}$ each with *nm* entries, where ${f}_{i}^{(j)}$ denotes the *i*-th component of the *j*-th feature vector. In our simulations we fixed *n* = 8 and $m=45$ for all models.

- Third, the binary feature vector *f* is translated into a scalar activation level ${g}_{\theta }(f)$. The function ${g}_{\theta }(\,\cdot \,)$ is different for each model type and the model-specific parameters θ can be adapted to emulate subjects’ adaptation processes. The different functions ${g}_{\theta }(\,\cdot \,)$ for the different models are specified below.
- Fourth, the activation level is transformed into an action probability by a soft activation function $\sigma (x)$, which we set $\sigma (x)=\frac{1}{\pi }{\rm{a}}{\rm{t}}{\rm{a}}{\rm{n}}(\varrho x)+\frac{1}{2}$ for all models. The gain parameter $\varrho$ is fitted for each model class separately and belongs to the hyper-parameters $\vartheta$ that are kept fix over the entire simulation.

In order to compensate for the choice of the human player’s minimal refractory period (see Supplementary Table S1), simulated units are only allowed to make decisions at a fraction $1-q$ of points in discrete time. We have fitted the parameter *q* such that the amount of players that are active at every point in time is a binomially distributed random variable with $q\,=\,\mathrm{19.39 \% }$, thus matching the distribution of coincidences in human subjects. Moreover, to make sure that our simulated decision-makers cannot become completely silent and inactive, in particular at the beginning of the learning process when only a few biased samples are available, we assume that units can randomly decide to press their buttons with probability $\varepsilon$ independent of the current cursor position. In the simulations we assume that $\varepsilon$ decays rapidly for spiking players (i.e. $\varepsilon =\,\max ({\beta }_{2},{e}^{-\rho c})$ with $c$ being the number of spikes produced by a player and $\rho =\,\log \,\mathrm{(0.85)}$. ${\beta }_{2}$ is a lower bound for $\varepsilon$ and a component of the hyper-parameters $\vartheta$. Mathematically, we can express the random decisions by mixing in the uniform distribution^69^. In summary, we can describe each decision unit by a probability distribution

$${P}_{\theta ,\vartheta }(a\,=\,1\,|{{s}}_{t},{t})=(1-q)\left(\frac{1}{2}\varepsilon +\mathrm{(1}-\varepsilon )\sigma ({g}_{\theta }({\rm{f}}(\phi ({t}-{{s}}_{t}))))\right),$$

$${P}_{\theta ,\vartheta }(a\,=\,0|{{s}}_{t},{t})\,=\,1-{P}_{\theta ,\vartheta }(a\,=\,\mathrm{1|}{{s}}_{t},{t}\mathrm{)}.$$

After an action $a$ has been sampled from ${P}_{\theta ,\vartheta }(a|{s}_{t},\tau )$ and the consequence in form of a cursor displacement ${{s}}_{t+1}-{{s}}_{t}$ has been observed, each decision unit updates its model parameters ${\theta }_{t}\to {\theta }_{t+1}$. In order to include the possibility of imperfect learning due to forgetting or inattention, decision units ignore the parameter update with probability $1-{\beta }_{1}$. The hyper-parameters $\vartheta$ are fixed for the course of the entire experiment. In the following we specify ${g}_{\theta }(\,\cdot \,)$ and *θ* for the different model types. The parameters are listed for reference in Supplementary Table S2.

#### Model 1: Bayesian decision-making

For the Bayesian model the activation level *g*_*θ*_ is given by the expected utility

11 $${g}_{\theta }(f)={\int }_{0}^{2\pi }{\int }_{0}^{{\rm{\infty }}}{\int }_{0}^{2\pi }{p}_{\theta }(\nu ,\kappa )p(\phi |f)\,U(a,\phi ,\nu )\,d\phi \,d\kappa \,d\nu ,$$

where we have the utility $U(a,\phi ,\nu )$ and probabilistic beliefs *p*_*θ*_ and *p*. The Bayesian decision-maker keeps an account of all possibilities and assigns probabilities to express their degree of plausibility. In our case, each decision unit needs to consider all the movement directions it could trigger and all the possible target difference vector angles that are compatible with the given feature vector *f*. We express the belief over displacement directions by the distribution ${p}_{\theta }(\nu ,\kappa )$ with displacement angle *v* and concentration parameter *k* and the belief over possible target difference vector angles by $p(\phi |{f})$. Learning in this model means adjusting the parameter vector θ. In order to reduce running time to a reasonable level we compute the cubic integral in Eq. (11) numerically in MATLAB^70^ by approximating the modified Bessel function of the first kind of order zero ${I}_{0}(\kappa )$ by its asymptotic equivalent ${\tilde{I}}_{0}(\kappa )=\frac{{e}^{\kappa }}{\sqrt{2\pi \kappa }}$ for large values of $\kappa$.

In our task, the utility is given by the reduction in distance to the target, which can be formulated in terms of the angular distance between the target difference vector angle $\phi$ and the displacement angle *v* such that

$$U(a,\phi ,\nu )=\{\begin{array}{cc}0 & {\rm{i}}{\rm{f}}\,{\rm{a}}=0\\ \frac{\pi }{2}-{d}_{C}(\phi ,\nu ) & {\rm{i}}{\rm{f}}\,{\rm{a}}=1\end{array}$$

with the circular distance function ${d}_{C}(\phi ,\nu )=\,\min \,\{|\phi -\nu |,\,2\pi -|\phi -\nu |\}$. To be able to evaluate this utility we need to reconstruct the target difference vector angle $\phi$ from the feature vector *f*, which is simply given by a uniform distribution over angles that lie in the intersection of all tiles of *f* with a nonzero entry, such that

12 $$p(\phi |f)=\{\begin{array}{cc}\frac{nm}{2\pi } & {\rm{i}}{\rm{f}}\,{d}_{C}(\phi ,{\bar{\phi }}_{f}) < \frac{\pi }{nm}\\ 0 & {\rm{o}}{\rm{t}}{\rm{h}}{\rm{e}}{\rm{r}}{\rm{w}}{\rm{i}}{\rm{s}}{\rm{e}},\end{array}$$

where ${\bar{\phi }}_{f}$ is the circular mean of all centers of the tiles that have a non-zero entry in *f*. For the displacement angle *v* the Bayesian decision-maker has to consider all possibilities, which we express by a von Mises distribution^71^

13 $${p}_{{\boldsymbol{q}}}(\nu ,\kappa )=\{\begin{array}{cc}\frac{{e}^{\kappa {R}_{0}\cos (\Phi -\nu )}}{K({R}_{0},c){I}_{0}{(\kappa )}^{c}} & {\rm{i}}{\rm{f}}\,\nu \in [0,\,2\pi ),\,\kappa \in {{\mathbb{R}}}^{+}\\ 0 & {\rm{o}}{\rm{t}}{\rm{h}}{\rm{e}}{\rm{r}}{\rm{w}}{\rm{i}}{\rm{s}}{\rm{e}}\end{array}$$

with parameters $\theta =({R}_{0},\varPhi ,c)$, normalizing constant $K(R,c)=2\pi {\int }_{0}^{\infty }\frac{{I}_{0}(R\kappa )}{{I}_{0}{(\kappa )}^{c}}{\rm{d}}\kappa$ and the modified Bessel function of the first kind  of order 0, i.e. ${I}_{0}(\kappa )=\frac{1}{\pi }{\int }_{0}^{\pi }{e}^{\kappa \cos (t)}dt\mathrm{}.$ The core of the Bayesian model update consists of adapting the parameters ${R}_{0},\Phi$ and *c* in the following way

14 $${R}_{0}\leftarrow \sqrt{{({R}_{0}\sin (\Phi )+\mathop{\sum }\limits_{j=1}^{n}\sin ({\phi }_{j}))}^{2}+{({R}_{0}\cos (\Phi )+\mathop{\sum }\limits_{j=1}^{n}\cos ({\phi }_{j}))}^{2}}$$

15 $$\Phi \leftarrow {\rm{a}}{\rm{t}}{\rm{a}}{\rm{n}}2({R}_{0}\,\sin (\Phi )+\mathop{\sum }\limits_{j=1}^{n}\,\sin ({\phi }_{j}),{R}_{0}\,\cos (\Phi )+\mathop{\sum }\limits_{j=1}^{n}\,\cos ({\phi }_{j}))$$

16 $$c\leftarrow c+k$$

where $\{{\phi }_{1}\mathrm{,..}.{\phi }_{k}\}$ are the angles of $k$ previous observations of cursor displacements made by the decision unit whenever it was active before (see Supplementary Fig. S3 for an illustration). In our simulations, all Bayesian decision units are initialized by ${R}_{0}=\mathrm{0,}\,\Phi =NaN$ and $c=0$.

#### Model 2: Thompson sampling

Thompson sampling is an efficient strategy to approximate the Bayes optimal decision in unknown environments, where the decision-maker samples from their beliefs and acts optimally given the sample. In our case of the Thompson sampling model, the activation level *g*_*θ*_ is given by a random sample of the expected utility

17 $${g}_{\theta }(f)=U(a,\phi {\rm{{\prime} }},\nu {\rm{{\prime} }}),$$

where $\phi {\prime} \sim p(\phi |{f})$ and $\nu {\prime} \sim {p}_{\theta }(\nu ,\kappa )$ from Eqs. (12) and (13), and $\theta =({R}_{0},\Phi ,c)$ as above. This way the expensive integration that is necessary for the Bayesian decision-maker is avoided and approximated, while also guaranteeing sufficient exploration due the sampling process. Moreover, since the beliefs are adapted just like in the Bayesian model, exploration is automatically reduced over time as certainty is gained. In our simulations, all Thompson sampling units are initialized by ${R}_{0}=\mathrm{0,}\,\Phi =NaN$ and $c=0$.

### Model 3: Reinforcement learning

In the reinforcement learning model, the activation level ${g}_{\theta }$ is given by the difference in the state action value function $Q({f},a)$ for the two actions $a=0$ and $a=1$, such that

18 $${g}_{\theta }(f)=Q(f,a=1)-Q(f,a=0),$$

where $Q(f,a)={{\bf{w}}}_{a}^{{\rm{T}}}f$ and the model parameters are given by $\theta =({\bf{w}})$ with $${\bf{w}}=(\begin{array}{c}{{\bf{w}}}_{0},{{\bf{w}}}_{1}\end{array})$$. The reinforcement learner updates the state action value function $Q({f},a)$ by trying to predict the future cumulative rewards given by the environment. In our task the rewards are given by ${r}_{t+1}=sign(\Vert {\rm{t}}-{{s}}_{t}\Vert -\Vert {\rm{t}}-{{s}}_{t+1}\Vert )$. Updating the action value function is achieved through SARSA($\lambda$), a standard model-free on-policy reinforcement learning algorithm). Model-free learning means associating the most rewarding actions with each occurring state without learning a predictive model of the environment, on-policy means that we use the same action value function for learning and action execution. Finally, $\lambda \in \mathrm{[0,}\,\mathrm{1]}$ is a forgetting parameter for a memory trace

$${e}_{t+1}(f,a)=\{\begin{array}{cc}1 & \,{\rm{i}}{\rm{f}}\,{f}_{t}=f\,{\rm{a}}{\rm{n}}{\rm{d}}\,{a}_{t}=a\\ \gamma \lambda {e}_{t}(f,a) & {\rm{o}}{\rm{t}}{\rm{h}}{\rm{e}}{\rm{r}}{\rm{w}}{\rm{i}}{\rm{s}}{\rm{e}},\end{array}$$

where we introduce again a combined variable $${z}=(e({{f}}^{\mathrm{(1)}},a=\mathrm{0),}\ldots ,e({{f}}^{(nm)},a=0),$$ $$e({{f}}^{\mathrm{(1)}},a=\mathrm{1),}\ldots ,e({{f}}^{(nm)},$$ $$a=1))$$ to represent the memory trace in vector form. For notational convenience^29^, we introduce the augmented feature vectors

$$\mathop{f}\limits^{ \sim }(a)=\{\begin{array}{cc}(\begin{array}{c}f\\ {\bf{0}}\end{array}) & {\rm{i}}{\rm{f}}\,{\rm{a}}=0\\ (\begin{array}{c}{\bf{0}}\\ f\end{array}) & {\rm{i}}{\rm{f}}\,{\rm{a}}=1,\end{array}$$

where ${\bf{0}}$ is a $nm\times 1$ dimensional vector of zeros. This allows for the following parameter update equation

19 $${{\bf{w}}}_{t+1}\leftarrow {{\bf{w}}}_{t}+\alpha {z}_{t}({r}_{t+1}-{{\bf{w}}}_{t}^{{\rm{\top }}}{\mathop{f}\limits^{ \sim }}_{t}(a)+\gamma {{{\bf{w}}}_{t}}^{{\rm{\top }}}{\mathop{f}\limits^{ \sim }}_{t+1}(a))$$

where the memory trace is replaced through ${{z}}_{t+1}\leftarrow \,\max ({{z}}_{t},{\tilde{{f}}}_{t}(a))$ before the update of **w** and decayed by ${{z}}_{t+1}\leftarrow \gamma \lambda {{z}}_{t}$ after the update. In our simulations, all reinforcement learning units are initialized by ${\bf{w}}={\bf{0}}$ and ${\bf{z}}={\bf{0}}$.

### Model 4: Perceptron

The perceptron (ANN) learning model with parameters $\theta =({\bf{w}},b)$ has the activation level

20 $${g}_{{\boldsymbol{q}}}({f})=\mathop{\sum }\limits_{j\mathrm{=1}}^{nm}{{w}}_{j}{f}_{j}+b,$$

where ${\bf{w}}$ is a $nm\times 1$-dimensional weight vector of a single-layer perceptron and $b$ is a scalar bias. A perceptron is a supervised binary classifier that receives a training signal $T\in \mathrm{\{0,}\,\mathrm{1\}}$ and assigns labels $y\in \mathrm{\{0,}\,\mathrm{1\}}$ to incoming data, which in our case is the feature vector $f$. The labels $1$ and $0$ correspond to pressing or not pressing the response button. The training signal is given by $T=H(\Vert \tau -{{\rm{s}}}_{t}\Vert -\Vert \tau -{{\rm{s}}}_{t+1}\Vert )$ where $H(\,\cdot \,)$ is the Heaviside step function. Accordingly, the training signal is either 1 or 0 depending on whether the cursor moved closer or farther away from the target position $\tau$. The update equations whenever the perceptron triggered a response are

21 $${\bf{w}}\leftarrow {\bf{w}}+\alpha (T-\mathrm{1)}{\bf{w}}$$

22 $$b\leftarrow b+\alpha (T-\mathrm{1)}b$$

such that no update takes place whenever label and training signal coincide or whenever the perceptron model unit stayed idle. In our simulations, all perceptron units are initialized by ${\bf{w}}={\bf{0}}$ and ${b}=0$.

The modification within the update equations is necessary in order to exclude a systematic error evoked by incorrect training signals. A generic example of this systematic error is the following: Every time a single unit is responsible for the cursor’s movement at a given point in time then at least two other units with similar displacement direction receive an incorrect training label. This effect leads to a negative net gain in knowledge across the group that eventually results in a degenerate behavior of all perceptron units.

## Supplementary information

Supplementary information.
