# Supplementary material for: Human group coordination in a sensorimotor task with neuron-like decision-making
Source: Sci Rep. 2020 May 19;10:8226. doi: 10.1038/s41598-020-64091-4 (PMC7237467; doi:10.1038/s41598-020-64091-4)
Supplement: Supplementary file 1 — Supplementary information. [file 41598_2020_64091_MOESM1_ESM.pdf]

Human group coordination in a sensorimotor task with neuron-like decision-making

Gerrit Schmid[1,*] **and Daniel A. Braun**[1]

[1]Faculty of Engineering, Computer Science and Psychology, Institute of Neural Information Processing, Ulm University, 89081 Ulm, Germany
[*]gerrit.schmid@uni-ulm.de

Supplementary material:

Figures

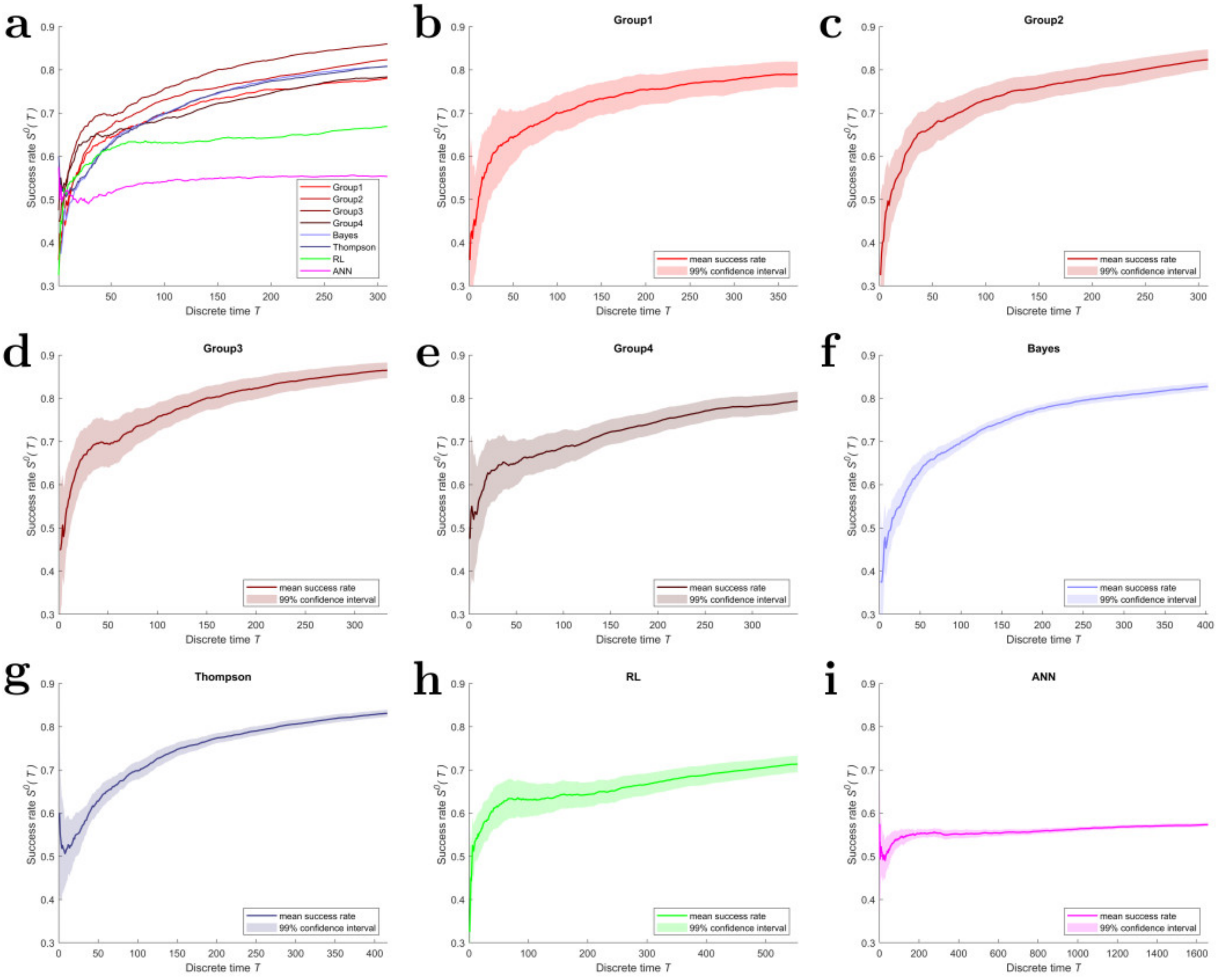

Figure S1. **Time-dependent success rates. a** Success rate as a function of time for the four human groups and the four computational models. **b-i** Success rate as a function of time for all individual groups. Shaded areas indicate 99% confidence intervals. The displayed time interval corresponds to the length of the shortest batch. Also note that the ordinate scale differs between panels.

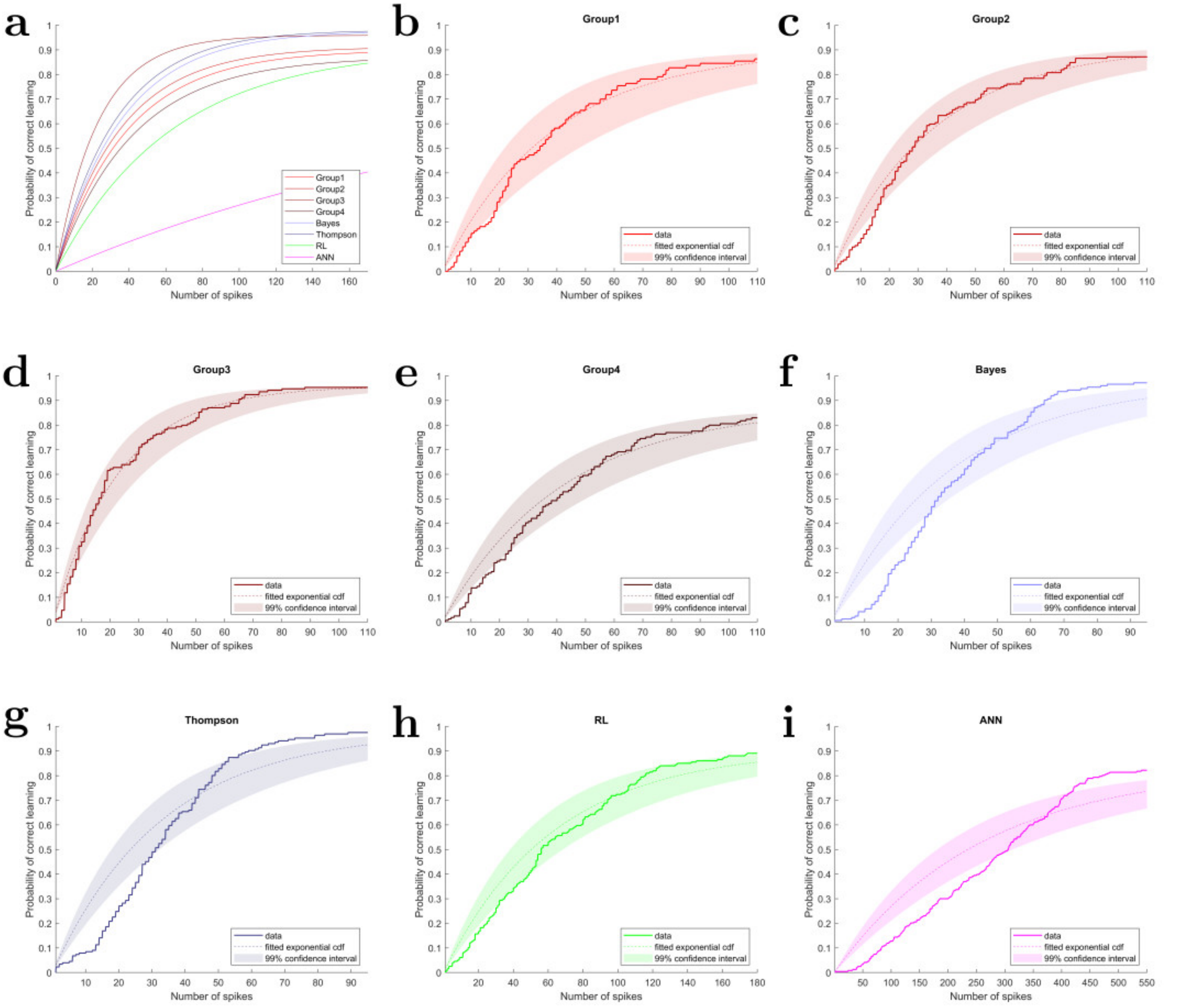

Figure S2. **Learning duration. a** Cumulative exponential fit to the learning duration of the different groups. **b-i** Proportion of batches with learning duration less or equal to the number of spikes averaged across all decision-makers for each individual group. Note that the scales are different between panels. Dashed lines indicate a cumulative exponential fit, the shaded area corresponds to the 99% confidence interval of the fitted parameter value.

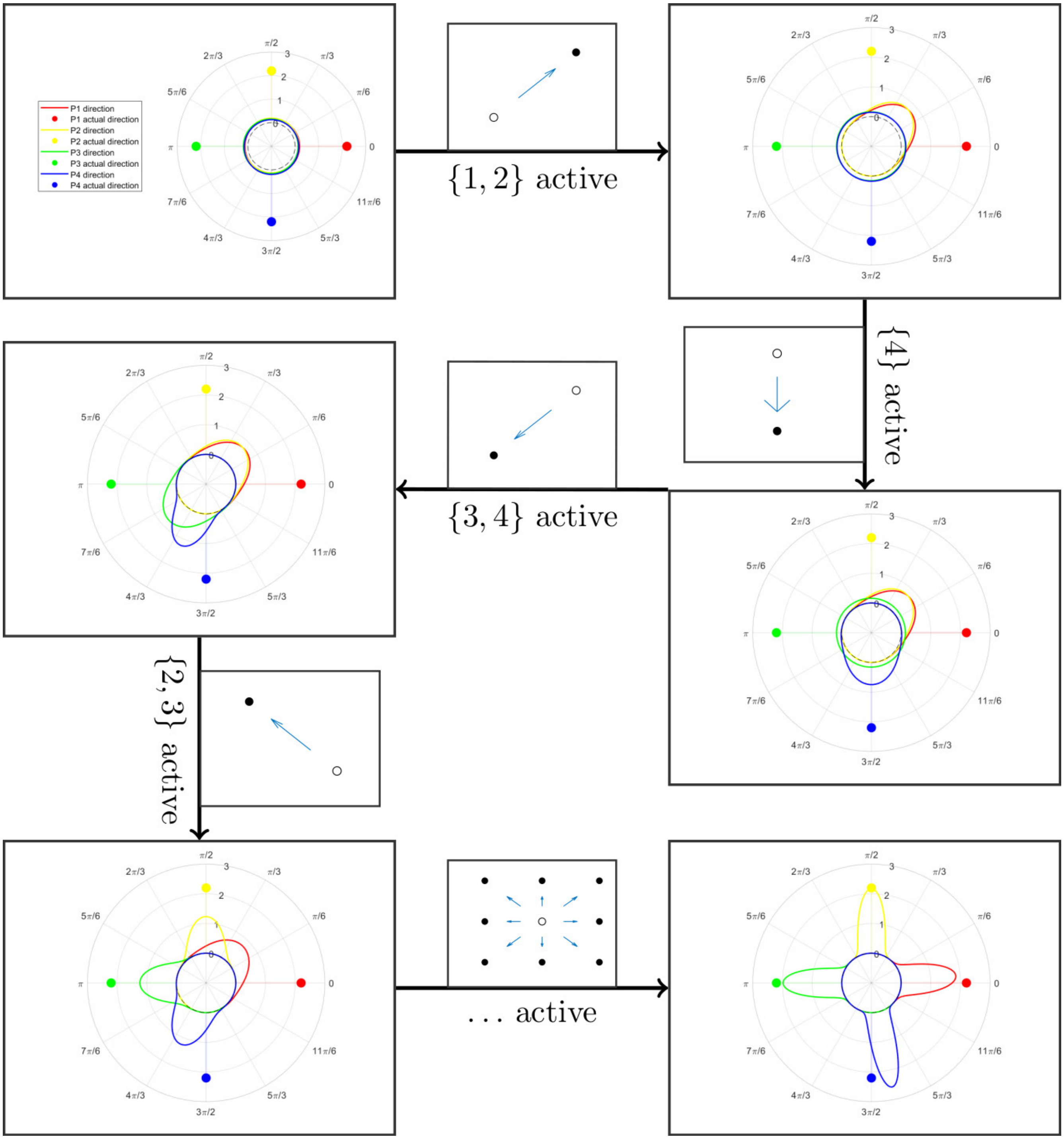

Figure S3. Bayesian inference process. Illustration of the Bayesian inference process of four players showing how the players incorporate new information in their direction. For the sake of an example the portrayed process is not a real inference progress observed in the experiment, but one with highly exaggerated effect of a single observation.

Tables

Table S1. Refractory period. Players' minimal refractory period T in ms in the four groups G1-G4. T is the minimum over the players' shortest duration between consecutive actions.

	**G1**	**G2**	**G3**	**G4**
T	135 ms	132 ms	134 ms	134 ms

Table S2. Summary of parameter notation. Quantities that require to be real-valued vectors are written in bold, scalar quantities are normal-sized.

	Notation	Explanation
common hyper-parameters	ρ	a scalar that controls the slope of the sigmoidal atan2 decision function
	β_1	the probability that an observation made by an agent is integrated in his existing knowledge
	β_2	a lower bound for the probability that actions are chosen randomly rather than greedily
	β_3	the standard deviation of Gaussian noise added to the observations
specific hyper-parameters	γ	discount-rate parameter for the SARSA learner
	α	step-size parameters for the SARSA and the perceptron (ANN) learner
	λ	decay-rate parameter for eligibility traces for the SARSA learner
setting	n,m	the amount of tiles per tiling $n = 8$ and the number of tilings $m = 45$ for the tile coding feature vector
	q	the probability that a unit can not decide whether to become active or not at a given point in time—fixed to $q = 0.1939$ and determined from the human players' data according to the choice of the players' minimal refractory period T
	ε	rapidly decaying parameter for an ε-greedy strategy initialized as 1 in order to force a random decision from all players at the beginning of every batch
	η	decay-rate parameter set to $\log(0.85)$ for the ε-greedy strategy with $\varepsilon_i \leftarrow e^{\eta c_i}$, with c_i being the number of spikes produced by player i
Bayesian model $\theta = (\Phi, R_0, c)$	Φ	circular mean of observations made.
	R_0	length of the vector sum of all observations.
	c	the number of observations made.
SARSA model $\theta = (w, z)$	w	$w = (w_1, \ldots, w_{2mn})$ - weights for every state-action pair
	z	$z = (z_1, \ldots, z_{2mn})$ - eligibility trace
Perceptron $\theta = (w, b)$	w	$w = (w_1, \ldots, w_{mn})$ - weights for every entry of the feature vector f
	b	scalar bias
